# The Immunoseasonal Theory of Psychiatric Disorders

**DOI:** 10.3390/jcm12144615

**Published:** 2023-07-11

**Authors:** Napoleon Waszkiewicz

**Affiliations:** Department of Psychiatry, Medical University of Białystok, Wołodyjowskiego 2, 15-272 Białystok, Poland; napoleon.waszkiewicz@umb.edu.pl; Tel.: +48-608-888-796

**Keywords:** psychosis, schizophrenia, depression, mania, bipolar, inflammation, weather, humidity, climate, immunoseasonal

## Abstract

Although the influence of the weather on the well-being and mental health of psychiatric patients has been widely seen, the relationships between various seasonal weather factors and depressive, manic, anxiety, and psychotic states have not been systematized in the literature. The current article describes the seasonal changes in weather-related immune responses and their impact on the development of episodes of depression, mania, psychosis, and anxiety, highlighting the T-helper 1 (Th1) and Th2 immune balance as their potential trigger. In autumn–winter depression, the hyperactivation of the Th1 system, possibly by microbial/airborne pathogens, may lead to the inflammatory inhibition of prefrontal activity and the subcortical centers responsible for mood, drive, and motivation. Depressive mood periods are present in most people suffering from schizophrenia. In the spring and summertime, when the compensating anti-Th1 property of the Th2 immune system is activated, it decreases the Th1 response. In individuals immunogenetically susceptible to psychosis and mania, the inhibition of Th1 by the Th2 system may be excessive and lead to Th2-related frontal and subcortical hyperactivation and subsequent psychosis. In people suffering from bipolar disorder, hyperintense changes in white matter may be responsible for the partial activation of subcortical areas, preventing full paranoid psychosis. Thus, psychosis may be mood-congruent in affective disorders.

## 1. Weather Factors and Psychiatric Disorders

An increasingly common position in the literature and media is that climate change and its resulting negative impacts on the physical environment can exacerbate poverty, malnutrition, and disease, including mental disorders [1]. The phenomena of seasonal variability in depressive, manic, and psychotic episodes have been described for a long time in psychiatry [2,3,4,5,6,7,8,9,10,11,12,13], but their interconnection has not yet been sufficiently explained. It has been suggested that seasonal mood disorders are caused by changes in atmospheric pressure and the accompanying baroreceptor reflex, and that hyperbaric oxygen therapy produces positive results similar to depression treatment with psychotherapy [9]. In recent years, researchers have also found a link between humidity and temperature and negative mental health effects, including suicide [12,13,14,15]. Increased temperature and vapor pressure have been associated with a significant increase in stress and anxiety, meaning that both can be modulated by humidity, as well as infection [13]. An analysis of meteorological factors, including atmospheric pressure, hours of sunshine, cloud cover, rain, fog, snow, air humidity, relative humidity, daily temperatures, and wind speed, found that relative humidity was the only factor that might influence depressive symptoms [15]. Weather conditions, such as temperature, wind patterns, and atmospheric pressure, affect humidity levels [14,15]. Warm air has the capacity to hold more moisture, resulting in a higher humidity, while cold air has a lower moisture-holding capacity, leading to a lower humidity. Additionally, wind patterns can contribute to changes in humidity by redistributing moisture across different areas. High humidity can impact human comfort levels. When the air is humid, it hampers the evaporation of moisture from our bodies, making it harder for sweat to evaporate and cool us down. This can result in feelings of stickiness, discomfort, and a perception of higher temperatures. Conversely, low humidity can cause dryness in the skin, eyes, and respiratory system, leading to discomfort and irritation [13,14]. Therefore, weather conditions have the potential to influence mood and emotions. While their specific effects vary from person to person, certain weather patterns have commonly been associated with mood changes. For example, sunshine and clear skies are often linked to positive moods, as sunlight promotes the production of the neurotransmitters (e.g., serotonin) associated with happiness and well-being [13,14]. Warm weather is generally associated with increased sociability, energy, and positive emotions [3]. Oppositely, rainy or cloudy weather when there is less daylight during the autumn–winter seasons can lead to feelings of sadness, lethargy, decreased motivation, hypersomnia, hyperphagia, and carbohydrate craving, which is called seasonal affective disorder (SAD) [2]. High humidity can also contribute to feelings of discomfort, which may indirectly impact mood. For some individuals, excessive humidity can lead to irritability, difficulty concentrating, and decreased motivation. Conversely, low humidity can cause dryness, which might also result in physical discomfort and affect mood negatively [13,14]. It is important to note that the influence of weather factors on humidity and mood can vary significantly based on individual preferences, geographic location, and personal experiences. Therefore, cultural factors and individual resilience also play roles in how people perceive and respond to different weather conditions [13,14]. Other research has indicated that seasonal temperature rises might induce a mood change from a depressive to a manic state [3], which may also be caused indirectly by changes in humidity [13]. The above mental health effects may also be modulated by changes in wind speed, air pressure, and air and water pollution [9,10]. A probable factor mediating the influence of wind and pressure on cognitive functions, anxiety, and mood, is their physical influence, through low-frequency waves, for example [9]. A significant intensification of symptoms and increased hospital admissions for people with schizophrenia and mania have been demonstrated during the spring and summer [3,4,5,6,7].

## 2. Immunology, Microbes, and Psychiatric Disorders

The role of immune system dysfunction in the pathogenesis of mental disorders has been described for many years [16,17,18,19,20]. Researchers have particularly looked at the balance between the T helper 1 (Th1) and Th2 immune responses in mental disorder development [17]. Pro-inflammatory cytokines, including interleukin-1 (IL-1), IL-2, IL-6, tumor necrosis factor-alpha (TNF-α), and interferon-gamma (IFN-γ), are mainly produced by Th1 lymphocytes, M1 monocytes/macrophages, and microglia [17,21]. Th1 cells are primarily responsible for the immune response to infections with intracellular bacteria and viruses, whereas M1 macrophages recruit and activate lymphocytes at the site of inflammation. The anti-inflammatory cytokines of the Th2 response are IL-4, IL-5, IL-10, and IL-13, which are mainly produced by Th2 lymphocytes, T regulatory cells (Tregs), monocytes/M2 macrophages, and astrocytes. Th2 lymphocytes are responsible for the humoral response and are active in allergic reactions and parasite infections. M2 macrophages are involved in wound healing, tissue repair, and remodeling after inflammation [17,21].

Unipolar depression (Major Depressive Disorder—MDD) has generally been found to stimulate a pro-inflammatory Th1 response (IL-1, IL-2, soluble IL-2 receptor (-sIL-2R), IL-6, C-Reactive Protein (CRP), TNF-α, and IFN-γ), with a subsequent increase in tryptophan catabolites (the kynurenine pathway) [17,22,23]. Cytokines can be produced by astrocytes and microglia in the brain or diffuse to the brain from the periphery through a leaky blood–brain barrier (BBB), active transport, endothelial cell activation, cytokine receptor binding, or via vagus nerve interaction in the gut, especially when cytokine levels are elevated [21]. An additional source of increased cytokine levels may be stress [17]. The hypothalamic–pituitary–adrenal axis (HPA) can be precipitated by exposure to stress, resulting in the release of glucocorticoid hormones, such as cortisol, which exert an inhibitory effect on immune cell cytokine production [24]. However, during chronic stress, its inhibitory effect on immunological functions can be lost, and increased cytokine levels can further suppress glucocorticoid receptors and contribute to persistent cortisol level elevation and cortex atrophy (e.g., the hippocampus) [24]. The pro-inflammatory cascade results in a subsequent decrease in excitatory amino acid activity, such as glutamates (GL), the abnormal modification and activity of dopamine (DA), serotonin (5HT), noradrenaline (NA), acetylcholine (ACh), and the kynurenine pathway [17]. Therefore, elevated pro-inflammatory cytokine levels reduce frontal brain activity [19], which may lead to symptoms of depression in immunogenetically susceptible patients [11]. Indeed, depression treatment significantly decreases the levels of some cytokines, including IL-1 [25], and treatment with anti-inflammatory substances, such as minocycline or N-acetyl cysteine (NAC) [22], may improve clinical outcomes in depression.

In bipolar disorder (BD), the findings regarding the individual cytokine concentrations in a specific disease phase are ambiguous [21,24,26,27,28]. In most cases, they indicate general Th1 lymphocyte activation in the course of BD, subsequent Th2 lymphocyte activation, and an increase in anti-inflammatory cytokines during mania, as opposed to an increase in pro-inflammatory cytokines during depressive episodes [21]. In bipolar depression, IL-6 seems to be the predominant cytokine, IL-4 dominates in the euthymic state, and IL-2, IL-4, and IL-6 are prevalent in the manic state, while patients with unipolar depression mainly have elevated TNF-α, IL-6, and sIL-2R [17]. Therefore, specific hypersomnia and a low level of psychomotor activity in BD might be due to the pattern differences or higher levels of some of these cytokines. Cytokine levels coincide with the peak periods of mania in spring and summer, when Th2-dependent allergies increase, and periods of depressive episodes in the winter and autumn, when several Th1-mediated infections are triggered [21]. Differences in the cytokine profiles between BD and MDD have also been confirmed by machine learning, in which higher levels of IL-10, IL-4, and thiobarbituric acid reactive substances (oxidative products—TBARS) have been distinguished between BD and MDD [28]. It has also been found that some drugs used as mood stabilizers (e.g., lithium) have immunomodulatory properties [26].

In schizophrenia, meta-analyses have supported the hypothesis of a decrease in Th1 immunity (IL-1 and TNF-α) in first-episode drug-naïve persons [29]. The typical changes in dopaminergic, serotonergic, noradrenergic, and glutamatergic neurotransmission described in schizophrenia patients have also been found in low-grade neuroinflammation, a key factor generating the symptoms of schizophrenia [29,30,31,32,33]. The importance of low-grade neuroinflammation in schizophrenia is supported by the loss of central nervous system volume and microglial activation demonstrated in neuroimaging studies. Finally, the benefits of anti-inflammatory drugs that have been found in some studies, such as celecoxib, and the anti-inflammatory and immunomodulatory effects of antipsychotics provide further support for the role of inflammation in this devastating disease [32].

Elevations in cerebrospinal fluid (CSF) cytokines suggest the possibility of glial immune activation in psychiatric disorders [20]. Meta-analytical schizophrenia data have found that the blood and CSF levels of IL-1β, IL-6, and IL-8, but not IL-2, were increased. Furthermore, the CSF sIL-2R levels were decreased, while its blood levels were increased [20]. In BD, increased blood and CSF IL-1β levels have been noted. The blood IL-6 levels were high in BD, but the CSF IL-6 levels were non-significantly lower than controls. Evaluations of MDD have found increased blood and CSF IL-6 levels and non-significant alterations in IL-1β, though the blood and CSF IL-8 and TNF-α levels were inconclusive. Schizophrenia studies have found higher IL-6 levels in the CSF than in the blood and lower IL-1β CSF levels compared to those in the blood, as well as lower levels of IL-1β, IL-6, and TNF-α and higher IL-8 levels in the CSF than in the blood of MDD subjects. However, the blood and CSF levels were not correlated for any cytokine [20]. As most CSF cytokine findings are generally based on a small number of studies/subjects and independent studies, measurements of peripheral blood cytokines and tryptophan catabolites may not necessarily reflect the immunological activity in the brain [20].

The immunological reactions in psychiatric disorders may be the result of microbial infection [21]. Some microbes have been associated with schizophrenia and other psychiatric disorders, such as BD, MDD, and autism spectrum disorders (ASD), including influenza viruses, human endogenous retroviruses, cytomegalovirus, Epstein–Barr virus, herpes simplex virus, and bacterial streptococcal infections in obsessive compulsive disorders (OCD) [34]. In addition, schizophrenia has been associated with intracellular bacteria such as chlamydia and the protozoan organism *Toxoplasma gondii* [34].

## 3. Immunoseasonal Theory of Psychiatric/Mental Disorders

The highest incidence of seasonal depression and catarrhal infections in the autumn–winter and early spring periods may be due to the link between depressive symptoms and viral infections due to changes in humidity [12,15,16]. It is possible that changes in humidity not only favor the airborne transmission of pathogens, but also cause condensation and subsequent body entry through drinking water, for example [15,16]. Such viruses include noroviruses, which are small enough and are not captured by water filters [16]. Infections caused by them reoccur seasonally and result in mild inflammation, such as oligosymptomatic infections starting in the gastrointestinal tract [16]. It is also known that some enveloped viruses, such as influenza and Respiratory Syncytial Virus (RSV), which contain a lipid membrane, survive better in a lower temperature and related humidity (RH) due to the higher ordering of the lipids in these conditions, while nonenveloped viruses tend to be more stable at a higher RH and temperature [35]. On the other hand, a high autumn humidity enhances indirect virus transmission by increasing a virus particle’s stability inside droplets and on surfaces, whereas the solar UV radiation of all wavelengths in spring–summer time can inactivate RNA and DNA viruses [35]. Therefore, hyperactivated Th1 immunity by viruses in wet autumn/winter/early spring weather seems to be not needed more in spring/summer time [35].

**Figure 1:**
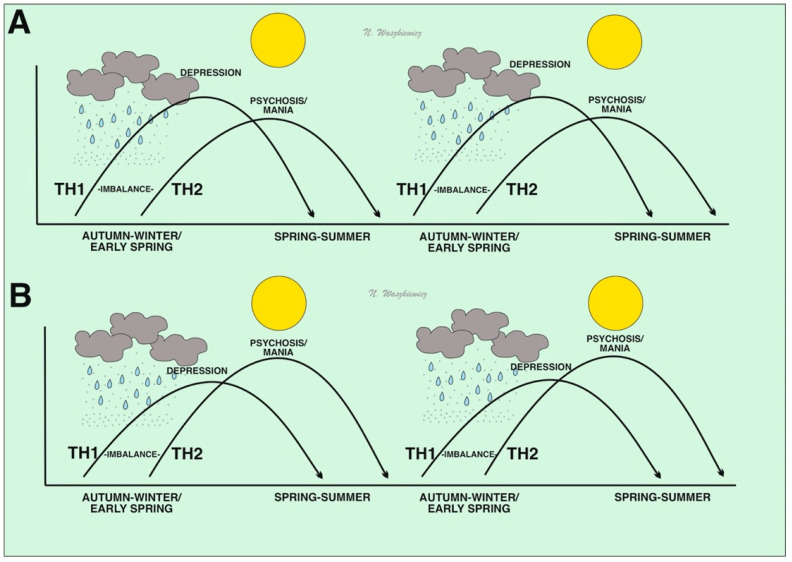
Disturbances in the balance of the Th1–Th2 immune response in people immunogenetically prone to depression (**A**) and schizophrenia/mania (**B**). In the figure, greater susceptibility to hyperactivation of the Th1 system may result from viral infection in the autumn–winter/early spring season, when there is higher humidity and a higher risk of pathogen droplet transmission or consuming pathogens by drinking condensed water-containing viruses. These events may result in a depressive phenotype. In spring and summer, when humidity and pathogen loads decrease, compensatory Th2 properties are activated. In individuals immunogenetically susceptible to psychosis and/or mania, Th2 inhibition of the Th1 system may be excessive and lead to Th2 hyperactivation and subsequent manic/psychotic states.

**Figure 2:**
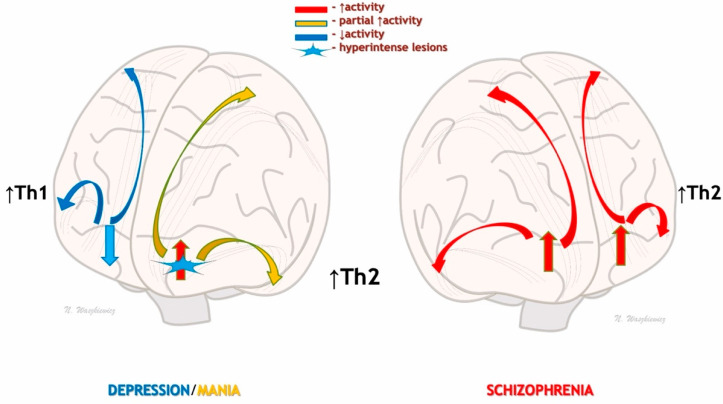
Disturbances in cortical activation depending on the activity of Th1 and Th2 immune responses in depression, mania, and schizophrenia. In autumn–winter depression, hyperactivation of the Th1 system may lead to inflammatory inhibition of prefrontal activity and subcortical centers responsible for mood, drive, and motivation (**left side**). During the spring–summer predominance of Th2 activity, there may be hyperactivation of the prefrontal areas and secondary hyperactivation of the subcortical centers responsible for mood and drive, resulting in psychotic/paranoid symptoms (**right side**). In people undergoing a manic episode (**left side**), hyperintense changes in the white matter may be responsible for the partial activation of subcortical centers and prevent full paranoid psychosis. Thus, psychosis may be mood-congruent.

Humidity can vary widely in different parts of the world, depending on the water proximity, temperature, and altitude [11]. However, it seems that changes in relative humidity, rather than its absolute value, are more important in recurrent seasonal infections, causing later immune changes and their consequences, including mental alterations [11]. According to the “sickness behavior” theory, symptoms of seasonal catarrhal infection (and/or flu) have similar features to depressive symptoms, such as weakness, sadness, anxiety, a lack of appetite, hypersensitivity, and problems with concentration, sleeping, and anhedonia, along with a tendency for isolation [17,18]. In evolutionary psychiatry, it is assumed that anhedonia allows for an infected organism to isolate itself from infecting other organisms. Moreover, the energy saved in this way can be used for self-healing [18]. Therefore, it is possible that an increase in infection caused by a potential pathogen is due to the weather changes (e.g., in air humidity) in autumn and winter and may be spread more easily through droplets [16], stimulating a Th1 pro-inflammatory response (IL-1, IL-6, and TNF-α) [17,22,23,24,25,26,27,28,29,30,31,32,33,34,35,36,37,38,39,40,41,42] (Figure 1A). These pro-inflammatory cytokines may reduce frontal brain activity, which can be observed in functional magnetic resonance imaging (fMRI) [19,25], and lead to depression symptoms [19] (Figure 1A and Figure 2). In severe depression, a lower mood may be related to the levels of some Th1 interleukins (IL-1 and TNF-α), but when depression lasts long enough, a simultaneous activation of the Th2 response and an increase in IL-6 [40] can lead to mood-congruent psychosis.

Mood drops do not only occur in people at an increased risk of depression and BD (affective immunophenotype), but also in those susceptible to schizophrenia (psychotic immunophenotype) [36]. Depressive episodes occur in up to 80% of people suffering from schizophrenia, and subclinical symptoms of depression are much more common [36]. Depressed mood in individuals with psychosis can trigger a pro-inflammatory (antiviral) Th1 response [17]. It appears that the body activates the anti-inflammatory Th2 immune responses that limit Th1 immunity to maintain the Th1–Th2 balance during an increased Th1 response. As such, hyperactivated Th2 responses can dominate during the spring–summer time, while Th1 declines (post-infection period; Figure 1B). Thus, it seems that pro-inflammatory Th1 cytokines, secreted mainly by microglia [16], may induce an astroglial-initiated [17] counterbalance and produce a Th2 immune response.

The Th2 response is hyperactivated during parasitic infections and allergic reactions [17,37,38]. Although allergic diseases often coexist in schizophrenia [36], the parasites are not identified, and only the anti-inflammatory (anti-parasitic) Th2 response and its related antibodies can be found (e.g., against *Toxoplasma gondii*) [38]. As such, the search for a parasite as the etiological cause of schizophrenia seems to be unfounded, as we would rather obtain the results of the previously acquired and currently hyperactivated Th2 immunity. Nonetheless, allergic and parasitic diseases that are more common in the spring and summer [37,38] may function as additional seasonal triggers of Th2 hyperactivation. In persons susceptible to psychosis (psychotic immunophenotype), Th2 hyperactivation can potentially result in an excessive glutaminergic stimulation of the frontal and mesolimbic cortical centers (e.g., via white matter bundles; Figure 2), as well as the modulation of other neurotransmitters (e.g., DA) and numerous pathways (e.g., with the formation of kynurenic acid having NMDA receptor antagonism), resulting in psychosis [17]. Moreover, it has been shown in fetal rat brains that higher cytokine levels in schizophrenia could promote mesencephalic progenitor cell conversion into a dopaminergic phenotype or decrease the survival of serotonergic neurons [32].

In contrast to the highly symptomatic paranoid psychosis observed in schizophrenia (thought echo/insertion/withdrawal/broadcasting, delusions of control, influence, and hallucinatory commentary/discussing voices) [32], a lesser symptomatic affective psychosis (less destructive psychosis) may result from a local inflammatory obstacle in the spread of the psychotic nerve activation from the frontal cortex to the lower subcortical parts of the brain (Figure 2). In the manic state of BD (increased self-esteem/grandiosity, high psychomotor activity, decreased need for sleep, logorrhea, distractibility, and risky, pleasurable behaviors), there is a hyperactivated Th2 response similar to that of schizophrenia [17,27]. Moreover, numerous similarities have been observed in genetic studies on schizophrenia and BDs, including immune-regulating genes such as the CCR chemokine receptor [42]. Concurrently, hyperintense changes in deep white matter and subcortical gray matter have been found in BD [43]. According to a meta-analysis, these hyperintense changes occur 2.5 to 5.7 times more often in people suffering from BD than the general population [43]. Indeed, they are observed in children and adolescents and 60% of healthy family members of bipolar persons [43]. Hyperintense changes in the prefrontal white matter that obstruct the conduction pathways may partially block the hyperstimulation of the subcortical mesolimbic areas and may be the reason why manic psychosis is limited to mood-congruent symptoms [11].

## 4. Stress Axis Pathway

Anxiety and the related activation of the HPA stress axis are associated with atmospheric and seasonal changes and occur in most (even up to ~80%) episodes of mood disorders and schizophrenia [8,11]. This is in line with recent findings that the Th17 immune response, which is linked with anxiety, can also promote the production of IL-6 and tumor growth factor (TGF), which dominate in depressive and psychotic states, respectively [17,39]. HPA axis cytokines can exert mood-affecting properties by inhibiting neurogenesis/synaptogenesis [11]. Since inflammatory cells use oxidative stress to fight infection and their values correlate with each other, oxidative stress may magnify cytokine-mediated brain damage [11]. These cytokines are also influenced by environmental factors and toxins [39].

## 5. Seasonal Immunity and Neurodevelopmental/Neurodegenerative Theories of Mental Disorders

The current neurodevelopmental and neurodegenerative theories of mental disorders [44,45,46] describe a greater brain tissue atrophy in schizophrenic psychosis than in affective disorders. This may be due to the tendency of the pro-inflammatory Th1 state to reduce the activity of the frontal areas [19], which leads to depression, but will not cause such a high neuronal apoptosis and cortical atrophy as that seen in paranoid psychosis. In schizophrenia induced by Th2 immunity [41], when psychosis is not significantly limited by hyperintense changes as in that in bipolarity [43], such a response may lead to much higher neurotoxicity. In MDD, cognitive impairment is temporally limited [17]. In schizophrenia, there is quite specific cognitive dysfunction that covers disturbances in working memory, attention, executive functions, information selection, and verbal fluency [41]. This cognitive decline is called an endophenotype, or even a marker of schizophrenia, and is a permanent feature in the family members of sick persons [41]. Cognitive functions gradually worsen over time in schizophrenia [41], which confirms that paranoid psychosis is much more neurotoxic than affective psychosis. Perhaps the immunogenetic profile of schizophrenia is responsible for this deterioration of cognitive function and cognitive gating problems [41]. Certain areas of the brain experience atrophy in psychotic, affective, and anxiety disorders and can undergo initial compensatory hyperactivation [47]. In schizophrenia, the initial cortex hyperactivation and lack of stimulus selection are a rather chaotic state of psychosis called disorganization [37,48]. Disorganization occurs to varying degrees in all persons with schizophrenia, and 93% of sick people show a moderate to severe intensity [48]. It may lead to gradual neurotoxic damage, which is especially intensified during acute psychosis.

## 6. Is It a New Psychiatric Episode or a “Flashback” Effect?

When psychotic and affective symptoms reoccur in susceptible individuals, they may be the result of a “flashback” effect [11]. We can imagine that, if potentially infectious or toxic pathogens can accumulate in the body, such as some psychoactive substances or viruses, the symptoms induced by them may occur without re-exposure [11]. This is the case in phencyclidine intoxication and varicella-zoster virus infection [11]. Even a long time after the pathogen enters the body, it can re-enter the bodily fluids. For example, phencyclidine can accumulate in fatty tissue after ingestion and enter the blood while burning fat [49]. The chickenpox virus resides in nerve endings and can be activated during immunodeficiency to produce shingles symptoms [50]. Potential schizophrenia and affective disorder pathogens could also use this latent flashback mechanism under sensory deprivation or inflammation conditions to inhibit the cognitive control of the prefrontal region and lead to an inhibition of the subcortical centers or their secondary psychotic hyperactivation [11].

## 7. Conclusions and Future Directions

Future neuro-immune-infectious research could help to elucidate whether the mechanisms of tissue accumulation and pathogen release in psychiatric disorders are viable and how they can activate or inhibit the cortico-limbic pathways. Perhaps they will also help us to look for a potential pathogen (microbial and/or airborne) that causes seasonal changes in the psyche and their relationship with weather factors such as humidity. As in the case of autoimmune encephalitis, which, until recently, was a mysterious and unexplained disease characterized by catatonic symptoms and disturbances in consciousness, psychosis, and agitation [51], much more common mental disorders also need a detailed medical explanation of their symptoms. Future studies should explore air humidity as a serious mind-changing factor alongside other factors, such as light and temperature, and investigate if humidity and/or temperature can change the prevalence of psychiatric disorders or their episodes in the world. It is also important to isolate the specific immune factors responsible for recurrent depression, manic, or psychotic states. Hence, there is an urgent need to find markers for mental disorders and answer the question of whether the Th1–Th2–Th17 immune interaction may predispose people to mental disorders during brain development and correspond to the neurodevelopmental and neurodegenerative theory. It is also worth looking for potential markers of mental disorders at a time when the activation of the stress axis is reduced to increase their specificity as much as possible. Future research should also show whether the immunoseasonal theory of mental disorders is true [52]. Since the current research on immune dysfunction in mental disorders mostly relies on peripheral markers, it would be beneficial to conduct more research based on central markers. However, it may be methodologically unattainable.

## Data Availability

Not applicable.
